# 50 years of hormonal contraception—time to find out, what it does to our brain

**DOI:** 10.3389/fnins.2014.00256

**Published:** 2014-08-21

**Authors:** Belinda A. Pletzer, Hubert H. Kerschbaum

**Affiliations:** ^1^Department of Psychology, Paris-Lodron-University SalzburgSalzburg, Austria; ^2^Center of Neurocognitive Research, Paris-Lodron University SalzburgSalzburg, Austria; ^3^Department of Cell Biology, Paris-Lodron-University SalzburgSalzburg, Austria

**Keywords:** hormonal contraceptives, synthetic steroids, progestins, androgenicity, ethinyl estradiol

## Abstract

Hormonal contraceptives are on the market for more than 50 years and used by 100 million women worldwide. However, while endogenous steroids have been convincingly associated with change in brain structure, function and cognitive performance, the effects of synthetic steroids contained in hormonal contraceptives on brain and cognition have barely been investigated. In this article we summarize the sparse findings, describing brain structural, functional and behavioral findings from the literature and suggest that synthetic steroids may contribute to masculinizing as well as feminizing effects on brain and behavior. We try to identify methodological challenges, explain, how results on endogenous steroids may transfer into research on hormonal contraceptives and point out factors that need to be controlled in the study of hormonal contraceptive dependent effects. We conclude that there is a strong need for more systematic studies, especially on brain structural, functional and cognitive changes due to hormonal contraceptive use. The hormonal contraceptive pill is the major tool for population control. Hence, such behavioral changes could cause a shift in society dynamics and should not stay unattended.

## Introduction

Hormonal contraceptive pills are on the market for more than 50 years (see Box 1). Today, about 100 million women worldwide use combined oral contraceptives (Petitti, 2003). However, despite the well characterized impact of endogenous female sex hormones on structure and physiology of the brain and their impact on cognition and mood, very little is known about the impact of synthetic steroids on cognitive abilities, brain structure and function. The impact of hormonal contraceptives on the brain is of great importance equally to the private user and physicians as well as to clinical and basic research. The far reaching consequences of hormonal contraceptives are illustrated by a few contrasting facts. (i) Kinsley and Meyer notice that the intake of steroids by an athlete is considered as abuse and “doping” by androgens is strongly sanctioned by society. On the contrary the intake of steroids in women is recommended for birth control around the world (http://www.scientificamerican.com). (ii) Adolescent girls start taking hormonal contraceptives earlier and earlier, often shortly after onset of puberty (Parkes et al., 2009). However, the majority of research on steroid actions in the brain focuses on post-menopausal hormone replacement therapy. (iii) Traditionally, medical as well as psychological research focused on male participants, because hormone fluctuations throughout the menstrual cycle were suspected to affect the results—rightly, as it turned out. Nowadays, numerous women participate in scientific studies. However, while participants on medication are excluded, studies hardly control for the use of hormonal contraception.

Box 1 **A short history of hormonal contraception.**

Within about a century, physicians, biologists, and chemists around the world elucidated the physiology of the ovary, manipulated its function, and triggered a global experiment, which influenced our society. At the end of the 19th century, anatomists concluded from histological sections that the corpus luteum in the ovary is a gland secreting a factor into the blood relevant to pregnancy (Frobenius and Fraenkel, 1999). In the early 1920s, the Austrian physician, Ludwig Haberlandt (1885–1932), published his findings on temporary sterilization of the female body. He realized that transplantation of ovaries from pregnant to non-pregnant animals prohibits pregnancy in the receiver. In a translational approach, Haberlandt suggested hormonal contraception also in women and collaborated with the company of G. Richter in Budapest (Hungary) to develop contraceptives for women (Haberlandt, 2009). In 1923, Edgar Allen and Edward Adelbert Doisy (1893–1986) published a bioassay, which used the vaginal epithelium response in castrated rodents to identify the bioactive component among corpus luteum factors as well as other sex hormones (Frobenius and Fraenkel, 1999). In the 1930s, progesterone was isolated from glandular extracts independently by four research groups, and its chemical structure was characterized by the chemist Karl Heinrich Slotta (1895–1987) (Frobenius and Fraenkel, 1999; Hawgood, 2001). In 1944, the Germans, Bickenbach und Paulikovics, published that progesterone (20 mg, daily) suppresses ovulation in women (Ludwig, 2011). Because progesterone is metabolized during the entero-hepatical passage, synthesis of chemically modified gestagens was a major challenge. Carl Djerassi, an Austrian-American chemist (born in Vienna in 1923), synthesized norethindrone, the first oral contraceptive, in the early 1950s. In the late 1950s, the American biologist, Gregory Goodwin Pincus (1903–1967) proved in a large scale study that oral application of hormonal contraceptives prevents pregnancy in women. In 1960, the “Food and Drug Administration” in the USA legalized the first contraceptive (Enovid^®^), a combination pill containing 10 mg of the progestogen, norethynodrel, and 150 μg of the estrogen, mestranol. In 1961, the pill (Anovlar^®^, Bayer Schering Pharma AG) was legalized in Europe. The minipil, a progestine-only pill, was introduced into the market in the 1960s.

In this article we summarize the sparse findings indicating brain structural, functional and behavioral changes due to hormonal contraceptive use and try to give methodological impulses for future research. The interpretation of these changes are demanding and at the moment speculative. Although, endogenous female sex hormones have been convincingly associated with changes in brain structure and physiology—estradiol and progesterone modulate e.g., glutamatergic, GABAergic, serotonergic and dopaminergic transmission (see Sacher et al., this topic)—the neuronal targets of synthetic steroids are almost unknown. Changes in brain structure and chemistry cause changes in cognition, emotion and personality and consequently in observable behaviors. If a majority of women use hormonal contraception, such behavioral changes could cause a shift in society dynamics. Since the pill is the major tool for population control, it is time to find out what it does to our brain.

## Effects of hormonal contraceptives: “feminizing” or “masculinizing”?

The combined oral contraceptive pill (OC) typically contains 0.02–0.04 mg ethinylestradiol and varying levels of synthetic progestins, which can be classified by their generation. Progestins are a heterogenous class of synthetic steroids, which differ in their strength of binding to steroid receptors, the sex hormone binding globuline or to enzymes involved in the metabolism of endogenous steroids (e.g., Sitruk-Ware, 2006). Furthermore, they differ in their impact on blood glucose levels and the lipid profile (e.g., Sitruk-Ware, 2006). Importantly, older generation progestins (e.g., Levonorgestrel, desogestel, gestoden, norgestimat), are as derivates of 19-nortestosterone, a common anabolic steroid (Brueggemeier, 2006), able to activate androgen receptors and exert androgenic actions (Sitruk-Ware, 2006; Wiegratz and Kuhl, 2006). Newer progestins (e.g., dienogest, drospirenone) on the other hand, bind very specifically to the progesterone receptor and are anti-androgenic (Wiegratz and Kuhl, 2006).

In the following we describe several mechanisms that may explain the effects of synthetic steroids on brain and behavior. We refer to an effect as “*feminizing*,” if it enhances a structural or functional sexual dimorphism favoring women. We refer to an effect as “*masculinizing*,” if it reduces a structural or functional sexual dimorphism favoring men.

### Synthetic steroids—possible mechanisms of action

Since ethinylestradiol and synthetic progestins act on estrogen and progesterone receptors and OC reduce endogenous testosterone levels (e.g., Jung-Hoffman and Kuhl, 1987; Graham et al., 2007; Hietala et al., 2007), any hormonal contraceptive, irrespective of the progestin component, may show feminizing effects on brain and behavior.

However, hormonal contraceptives do also lead to a reduction of endogenous estradiol and progesterone levels (e.g., Sahlberg et al., 1987). In the presence of high levels of progesterone, testosterone-actions are impaired, because progesterone has a high affinity for the enzyme 5α-reductase, which is responsible for the conversion of testosterone into the physiologically more active dihydrotestosterone (Wright et al., 1983). If progesterone levels are reduced, more testosterone can be converted to dihydrotestosterone. Thus, any hormonal contraceptives, irrespective of the progestin component, may facilitate testosterone actions on the brain, thereby masculinizing brain structure, function and behavior. Alternatively, it has been argued that some masculinizing effects are promoted by estrogen receptors after testosterone has been locally converted to estrogen via the enzyme aromatase (Roselli, 2007). Consequently, estrogenic actions of ethinylestradiol may contribute to possible masculinizing effects of hormonal contraceptives on the brain.

### Androgenic vs. anti-androgenic progestins

Furthermore, the actions of synthetic progestins on the androgen receptors, may contribute to possible masculinizing and feminizing effects. While androgenic progestins may promote masculinizing effects, anti-androgenic progestins may promote feminizing effects. This distinction has important methodological consequences. The preferable approach to studying the effect of hormonal contraceptives in brain and behavior is a within-subjects design, i.e., comparing women to themselves, before, during and after the period of contraceptive use. Since this is however complicated, costly and time-consuming, hormonal contraceptive dependent effects are often studied by comparing a group of OC users to a group of non-users. If the group of OC users is heterogenous with respect to the androgenicity of the progestin component, possible feminizing and masculinizing effects may counteract each other, leading to results that either under- or over-estimate the actual effects and are hard to compare between different studies. While the first studies in this domain added important explorative impulses, by demonstrating that OCs may indeed influence brain and behavior, we strongly argue that the androgenicity of the progestin component is an important factor that should be controlled in future studies. An interesting observation from our own research experience in that respect may help when comparing findings between studies. We conducted two studies on the effects of OCs only about a year and a half apart, the first one in middle Europe (Austria; Pletzer et al., 2014a), the second one in the US (California, Pletzer et al., 2014b). While in the Austrian sample (Pletzer et al., 2014a), the group of OC users consisted of a majority of women using newer pills containing anti-androgenic progestins, in the US sample (Pletzer et al., 2014b), all OC users were on older pills containing androgenic progestins.

### Effects of hormonal contraceptives on brain structure

Adult brain structure is not static, but subject to dynamic changes with age. These changes do differentially affect different brain areas, i.e., gray matter volumes in some areas decline more strongly with age than others. A strong age-related decline has for example been demonstrated in the prefrontal cortex, as well as the hippocampus (e.g., Sowell et al., 2003).

Recent results demonstrated that regional gray matter volumes in the prefrontal cortex, as well as the anterior cingulate gyrus are larger in mixed samples of androgenic and anti-androgenic OC users compared to non-users (Pletzer et al., 2010; DeBondt et al., 2013). These regions are already larger in women compared to men (e.g., Good et al., 2001; Pletzer et al., 2010). However, regional gray matter volumes of OC-users were also larger in the cerebellum, hippocampi, parahippocampal and fusiform gyri (Pletzer et al., 2010; DeBondt et al., 2013). Those regions are on the average larger in men compared to women (e.g., Good et al., 2001; Pletzer et al., 2010). Results from rodent hippocampi suggest that these volume increases may be attributed to an increase in synaptic spine density mediated by estrogen receptors (e.g., Murphy et al., 1998; McEwen, 2002; Smith et al., 2009), but an increase in astrocyte volume in response to estradiol has also been suggested (e.g., Spencer et al., 2008).

Newer results from our own lab, suggest that the effects in cerebellum, parahippocampus and fusiform gyri are attributable to OCs containing anti-androgenic progestins, while the results in the prefrontal cortex are to be interpreted with care (Pletzer et al., submitted). We demonstrate that some structural effects increase with the duration of pill use, some interact with the age of the participants and some may not be completely reversible, and hence the duration of previous pill use in the group of non-users plays an important role. Thus, despite the androgenicity of progestins, the duration of (previous) pill use and age are important factors, that should be controlled when studying the effects of OCs on brain and behavior. OCs, in particular those containing anti-androgenic progestins, are commonly used by younger women, while naturally cycling women tend to be on average a few years older.

### Effects of hormonal contraceptives on brain function—neurocognitive findings

Synthetic steroids may however, not only cause a structural re-organization of the brain, but—even more importantly—induce changes in neurochemistry and brain function, which are currently relatively unexplored. Despite the relatively robust finding that OC users show altered mate preferences (e.g., Alvergne and Lummaa, 2009), accompanied by changes in brain activation patterns during viewing of erotic stimuli (Abler et al., 2013), OC dependent changes in cognitive performance have not been studied systematically.

Scattered over several countries and decades, it has been reported that OC users show enhanced verbal memory (Mordecai et al., 2008), recognition working memory during sleep deprivation (Wright and Badia, 1999), a lack of memory impairment due to cortisol (Kuhlmann and Wolf, 2005) and better dream recall (Sheldrake and Cormack, 1976) compared to non-users. These verbal abilities and memory are usually thought to favor women (e.g., Andreano and Cahill, 2009). However, non-significant effects were reported for other measures of verbal abilities, like verbal fluency (Mordecai et al., 2008) or a verbosequential task (Gordon and Lee, 1993), while verbal reaction times were slower in OC users compared to non-users in an older study (Garrett and Elder, 1984). First evidence for brain functional differences between OC users and non-users has also been reported in the verbal domain. A German brain imaging study (Rumberg et al., 2010) observed that during a word generation task OC users showed stronger activations in right-hemispheric task-specific areas than non-users. This is of particular interest, since the peak coordinates of the task-specific activations were left-lateralized in all participants and sex differences in lateralization have long been discussed in particular with respect to verbal tasks (e.g., Renteria, 2012).

On the other hand, some studies report better mental rotation performance in OC users compared to non-users (Wright and Badia, 1999; Wharton et al., 2008), although other studies report non-significant effects on visuospatial tasks (Gordon and Lee, 1993; Mordecai et al., 2008). Spatial abilities have robustly been observed to favor men (Andreano and Cahill, 2009). Wharton et al. (2008) nicely demonstrated that mental rotation performance does not only correlate with hormonal contraceptive use, but also with the androgenicity of the progestin component. Users of drospirenone-containing contraceptives performed worse on the mental rotation task than non-users. Neuroimaging studies exploring functional differences in spatial tasks between OC-users and non-users are still lacking. However, it has been demonstrated that brain activation patterns are masculinized in numerical tasks (Pletzer et al., 2014a), which have been related to spatial abilities (e.g., Hubbard et al., 2005).

Furthermore, OC users perform like men in an emotional memory paradigm, designed by Cahill and coworkers (Nielsen et al., 2011, 2013) and in a Navon paradigm (Pletzer et al., 2014b), both studies conducted on US samples. Brain functional differences between OC users and non-users have also been reported during the resting state (Petersen et al., 2014), during face processing (Mareckova et al., 2012) and during reward processing (Bonenberger et al., 2013). However, further more systematic studies are needed to reveal the true nature of OC-dependent effects on cognition as well as the impact of synthetic steroids on the neuronal correlates. Importantly, it has been demonstrated that endogenous sex hormones, in particular estrogen, affect cognitive performance differentially in different subpopulations of women, as some neurotransmitters (e.g., dopamine) affect behavior in an inverse U-shaped manner (Jacobs and D'Esposito, 2011; Colzato and Hommel, 2014). Hence, like in the study of menstrual cycle dependent effects (Colzato and Hommel, 2014), it might be worth considering neurotransmitter baseline levels, when studying the effects of hormonal contraceptives on brain and behavior.

### Effects of hormonal contraceptives on emotion

Reports on OC-related mood changes are inconsistent, ranging from beneficial in most women (Oinonen and Mazmanian, 2002) to increased rates of depression, anxiety, fatigue, neurotic symptoms, compulsion and anger (Robinson et al., 2004; Kulkarni, 2007). Repeated findings of beneficial mood changes may however be biased by data sampling. Only women, who continue the intake of oral contraceptives (“survivor effect”) are included in those studies, while women, who discontinue the use of oral contraceptives due to negative emotional side effects, do not contribute to these results (Oinonen and Mazmanian, 2002).

Possible physiological mechanisms underlying both positive and negative mood swings in oral contraceptive users are manifold and at the moment speculative. Elevated levels of estradiol have anti-depressive effects (Moses-Kolko, 2009; Estrada-Camarena, 2010), presumably due to its serotonin (5-HT) enhancing property (e.g., Bethea et al., 2002). A decline in estradiol at the end of the menstrual cycle, post-partum or in the menopause has been associated with negative mood changes and depressive symptoms during these phases (Moses-Kolko, 2009). On the one hand combined OCs contain ethinyl-estradiol as synthetic agonist for estradiol receptors, which could promote positive mood changes. Antagonistic properties for the 5-HT3 receptor have been demonstrated not only for estradiol, but also for ethinylestradiol (Wetzel et al., 1998). On the other hand the levels of endogenous estradiol decline as a consequence of OCs (e.g., Sahlberg et al., 1987), which could result in negative mood changes. In female cynomolgus monkeys on OCs a decreased prolactin response was observed, suggesting reduced serotonergic activity (Henderson and Shively, 2004). Progesterone, however, may promote positive mood changes at low concentrations and negative mood changes at high concentrations due to biphasic effects on GABAergic neurons (Andréen et al., 2009). Again, synthetic progestins as contained in OCs simultaneously act as progesterone receptor agonist and reduce the level of endogenous progesterone (Wright et al., 1983; Sahlberg et al., 1987). In a meta-analysis Oinonen and Mazmanian (2002) suggest that the progesterone/estrogen ratio correlates to the direction of emotional changes.

In summary, these results suggest that there are (at least) two populations of women with differential emotional responses to oral contraceptive use. Interestingly, the majority of studies focused on depressive symptoms, while other emotional and personality dimensions, like aggression or empathy, have hardly been investigated.

## Puberty and hormonal contraceptives

Progestin affecting either metabolism of neurosteroids or binding to GABA_A_ receptors may have not only transient but also neuroplastic consequences. We assume that the potential influence of progestins on GABAergic transmission is highly relevant in pubescent girls using hormonal contraceptives. (1) With onset of puberty, neurosteroid sensitive GABA receptor expression increases at extrasynaptic sites in female mice (Smith, 2009). (2) Enhanced GABAergic transmission shortens whereas depression of GABAergic transmission extends the critical period in structural consolidation of neuronal circuits in the visual cortex in mice (Hensch, 2005). (3) Final volume of the prefrontal cortex is not reached until the early twenties in humans (Yurgelun-Todd, 2007). The maturing of the prefrontal cortex is associated with improvement in cognitive abilities as well as behavioral control (Yurgelun-Todd, 2007). The prefrontal cortex appears to be one target of structural changes in hormonal contraceptive users (Pletzer et al., 2010; DeBondt et al., 2013). Accordingly, pharmacological intervention by the early use of hormonal contraceptives could affect the differentiation of neural circuits in the prefrontal cortex.

### Summary and conclusion

First and foremost, we conclude that there is a strong demand for additional studies on how hormonal contraceptives affect the brain from the molecular to the behavioral level. Thus, future studies aiming to investigate “normal” brain functioning, should control for the use of hormonal contraceptives among their participants. At both the structural as well as behavioral level, feminizing and masculinizing effects of hormonal contraceptives have been observed simultaneously. However, these changes may show differential manifestation at the behavioral level in different subpopulations of women. As the number of women using oral contraceptives constantly increases, while the age of first contraceptive use constantly decreases down to sensitive neuroplastic periods during puberty, the associated changes in personality and social behavior imply significant consequences for society.

### Conflict of interest statement

The authors declare that the research was conducted in the absence of any commercial or financial relationships that could be construed as a potential conflict of interest.
